# Prevalence of burnout and associated factors among midwives, 2023: institution-based cross-sectional study

**DOI:** 10.3389/fpubh.2024.1422915

**Published:** 2024-06-24

**Authors:** Solomon Seyife Alemu, Mohammedamin Hajure, Mahlet Tesfaye Agago, Feisal Hussein, Hana Israel Gesisa, Sheleme Mengistu Teferi, Daniel Yohanes, Lema Fikadu Wedajo

**Affiliations:** ^1^Department of Midwifery, College of Health Sciences, Madda Walabu University, Sheshemene, Ethiopia; ^2^Department of Psychiatry, College of Health Sciences, Madda Walabu University, Sheshemene, Ethiopia; ^3^Department of Midwifery, Institute of Health Sciences, Jimma University, Jimma, Ethiopia; ^4^Department of Midwifery, College of Health Sciences, Mattu University, Mattu, Ethiopia; ^5^Department of Midwifery, Institute of Health Sciences, Wollaga University, Nekemte, Ethiopia

**Keywords:** burnout, associated factors, midwives, health professionals, West Arsi, Ethiopia

## Abstract

**Background:**

One of the main characteristics of the mental health condition known as burnout syndrome is an overwhelming feeling of physical and emotional tiredness, particularly with regard to one’s work. Midwives are the group most prone to burnout because they work in emergency situations to save two lives at a time, share the stress of laboring women, and put in extra hours without enough payment. Besides this, there is little information on burnout among Ethiopian midwives.

**Objectives:**

To assess burnout and associated factors among midwives working in public health facilities in West Arsi Zone, Ethiopia.

**Methods and materials:**

A census method cross-sectional study was conducted among all 467 midwives working in public health facilities found in the West Arsi Zone, Ethiopia, from September 1 and 30, 2023. A pretested, validated face-to-face interviewer-administered structured questionnaire was used to collect data. Then, binary logistic regression was used for analysis. Bi-variable and multivariable logistic regression analyses were employed to identify factors associated with burnout. The level of statistical significance was declared at *p* < 0.05 with a 95% CI.

**Results:**

Overall, the prevalence of burnout among midwives was 47.10% (95% CI: 42.55, 51.75%). Marital status not in union 2.03 (95% CI: 1.32–3.13), working more than 40 h per week 2.00 (95% CI: 1.29–3.08), conflict with their metron 2.33 (95% CI: 1.54–3.54), not satisfied with their current job 2.39 (95% CI: 1.56–3.66) and having depression symptoms 1.71 (95% CI: 1.06–2.74) were factors significantly associated with burnout.

**Conclusion:**

This study found that in the study area, almost half of the midwives experienced burnout. Thus, it is recommended that midwives should develop respectful interactions with both their mentors and colleagues. Secondly, we suggest that zonal health offices set up systems that by shortening working hours and boost job satisfaction by creating conducive working environment, provide opportunities for career advancement and increase employee engagement.

## Introduction

Safe and healthy work environments can have a significant impact on the general well-being of individuals, including mental health. Apart from the economic advantages, it also helps reduce stress and arguments at work and enhance employee retention, production, and performance (1).

Globally, mental health issues are thought to cost the world $1 trillion annually in lost productivity. On behalf of this mental health disorder’s effect on a person’s ability to engage in social activities, perform well at work, and maintain connections with friends and coworkers (2). The majority of mental health issues are readily and affordably treatable, but if they are not identified and treated in a timely manner, they can have severe effects on a person’s socioeconomic status and other aspects of their life (2, 3).

Burnout syndrome is a mental condition that is characterized by an intense sense of physical and emotional weariness that an individual experiences, particularly in relation to their work. This condition is typically associated with first responders, business people, and healthcare practitioners. Low productivity, nervousness, tiredness, mental blockage, and widespread skepticism of one’s own work are its defining characteristics (4, 5).

Maternity care in particular is one of the most important components of the healthcare system, playing an essential function in the present as well as the future of society (6, 7). Working in this unit has a dual burden for health professionals as they provide basic healthcare as well as handling problems that come up during the course of pregnancy, childbirth, and the postpartum phase. Among all health providers, midwives typically handle problems and obstacles that are unique to women (8, 9).

Different literatures argue that midwives are suffering from a variety of mental health issues, including burnout, because of the stressful nature of their jobs (7, 10, 11). Globally, multiple studies showed that the magnitude of burnout among midwives varied from 20 to 59% (8, 9, 12, 13). The percentage is higher in low-income nations. For instance, a study done in Ghana (14) revealed that midwives had 65.8% burnout compared with the study conducted in the Netherlands (15), where it found that burnout was barely 7%. In sub-Saharan countries, particularly in Ethiopia, midwives often struggle with problems such as an excessive workload, a lack of resources for maternal health, and workplace hostility from clients and attendants (16–18). Furthermore, they receive inadequate salary and compensation for their efforts, a risk allowance, and non-regular time payments. Thus, for those various reasons, midwives have a greater risk of developing work-related burnout (19). A pocket of studies conducted in Ethiopia revealed that the magnitude of burnout among midwives ranged from 51.9 to 60.47% (16, 20).

The effects of burnout are diverse. Burnout among midwives has a great negative impact on maternal mortality and neonatal deaths in sub-Saharan African countries, including Ethiopia (21). For instance, according to a recent Ethiopia Demographic Surveillance report, there are 30 neonatal deaths and over 412 maternal deaths in Ethiopia (22). In addition to this, work-related burnout has numerous detrimental effects, including a decline in production, unhappy employees, professional disengagement, an increased risk of medical malpractice, decrease quality of maternity care, undesirable pregnancy continuum of care, a subsequent increase in staff turnover, and a higher intention to stop work (23). Additionally, it has an impact on their personal lives through social disengagement, anxiety, and hopelessness (24).

Some earlier research has found that variables like the sex of midwives, age of midwives, years of service, workload, resource availability, adverse patient outcomes, disagreements with coworkers, a lack of organizational support, a lack of recognition, and stressed-out work were significantly associated with burnout among midwives (23–25). Mental health conditions will become the leading cause of disability-adjusted life years (DALYs) by the 2030 year (26). Reports from the Compressive Mental Health Action Plan 2030 seek to enhance mental health (27). The World Health Organization changes this to “mental health for all” and exhorts nations to move quickly to put this plan into action (28). On the other hand, by using skilled delivery attendants, particularly midwives, mothers, and newborns, deaths are decreased by more than half and three-quarters, respectively (29, 30). In contrast to this, evidence in Ethiopia indicates that midwives have a high turnover rate and low job satisfaction, which has an impact on burnout (31, 32). While burnout among midwives has a wide range of impacts, ranging from an effect on personal life to a great contribution to maternal and neonatal death, the quality of maternity care, and others, not much is known regarding burnout among Ethiopian and other sub-Saharan African midwives. Determining the prevalence of burnout and its contributing factors among midwives in the West Arsi Zone in 2023 was the goal of this study.

## Methodology

### Study area, study design, and period

A census method cross-sectional survey was conducted among all midwives employed in public health facilities situated in the West Arsi zone between September 1 and 30, 2023. This zone is in the Oromia region of southern Ethiopia. The zone contains one reference hospital, four primary hospitals, two general hospitals, and 85 health centers. At these health facilities, 467 midwives and 450 other healthcare workers provided maternity and child health services, according to data gathered from the zonal health office. In 2023, the zone would be home to 647,690 women in the reproductive age group and 101,558 projected deliveries annually.

#### Study population

All midwives working in public health facilities found in West Arsi zone.

### Eligibility criteria

The study included midwives who had worked for a minimum of 6 months in public health institutions situated in the West Arsi zone; however, those who were on maternity leave in addition to annual leave were excluded.

### Sample size determination and sampling technique

All midwives working in public health facilities in the West Arsi zone of Ethiopia were included in this survey. There were 467 midwives working in 92 public health facilities in the West Arsi Zone, according to the zonal health office. Thus, all midwives at all healthcare facilities participated in this research.

To check the adequacy of the sample size, post-hoc analysis for power calculations was employed. Based on the following assumptions: Effect size f (medium): 0.25, α error probability: 0.05, total sample size: 467, number of groups: 2.

Thus, the result of power was 0.999, which suggested an adequate sample size.

### Study variables

#### Dependent variable

Burnout was the dependent variable.

#### Independent variables

##### Socio-demographic variables

Sociodemographic factors like age of study participant, marital status of study participant, and educational status of study participant were independent variables included in this study. Additionally, the residence of the study participant, religion, ethnicity, occupation of the study participant, and monthly income factors were seen as independent factors in this study.

##### Service, organization, and social related factors

Health care service-related as well as social-related factors like types of health facilities, years of service of study participants, work hours per week, history of chronic medical illness, conflict with coworkers, conflict with their metron, salary enough in the current job, availability of resources, job satisfaction, social support, and depression symptoms were included in this study.

### Operational definition

#### Burnout

To assess symptoms of burnout syndrome there were 19 items that were categorized into four Likert scales 0 (never), 1 (sometimes), 2 (often), and 3 (always). The sums of the scores of the rating of the items were calculated with a total minimum score of 0 to a maximum score of Healthcare. Obstetrics care providers that scored more than 23 out of 57 were considered to have burnout symptoms (33).

#### Job satisfaction

The job satisfaction was categorized into five Likert scales (from strongly disagree to strongly agree). Those Obstetrics care providers were considered as satisfied with their job if they answered greater than or equal to the mean value 50% (34).

#### Depression

The Patient Health Questionnaire-9, with their responses ranging from “0” (not at all) to “3” (nearly every day), was used for the assessment of depressive symptoms, with a total score of nine items ranging from 0 to 27. Based on the previous study, those Obstetrics care providers who scored ≥10 were considered to have depression symptoms, and a code “1” was given. While those who scored <9 were considered to have no depression symptoms, and a code “0” was given by Patient Health Questionnaire-9 tool (35).

#### Social support

Social support was used to assess social support. The OSSS-3 sum score then was operationalized into three broad categories of social support based the score as follows. Oslo Social Support Scale (OSLO) was used, and classified as 3–8 poor social support code “1”, 9–11 moderate social support code “2”, and 12–14 strong social Support code “3” was given (36).

### Data collection tool and procedure

The information was gathered via a previously validated questionnaire that was given out by interviewers. The questionnaires include questions about sociodemographic characteristics, aspects relating to services, social support, and organizations, in addition to questions about burnout, depression, and job satisfaction. The questionnaires to assess socio-demographic factors, service-related, and organization related factors were developed from previous published literature. The tool to assess symptoms of depression symptoms (35), job satisfaction (34), and social support (36) was validated in Ethiopia.

Burnout was the outcome variable. Contains 19 items that are divided into four Likert scales: 0 (never), 1 (sometimes), 2 (often), and 3 (often) to evaluate burnout syndrome symptoms. With a total minimum score of 0 and a maximum score of Healthcare, the sums of the ratings of the elements were computed. Providers of obstetric care who scored higher than 23 out of 57 were deemed to have symptoms of burnout (33). The questionnaire was created in English and then translated into Afan Oromo and Amharic to ensure uniformity. Data collection was conducted using the English language. Twenty supervisors and forty-five data collectors were hired for the data collection process. Supervisors oversaw the data collection process daily.

### Data quality control

A pretest was conducted on 10% of the sample size of midwives working in East Bale Zone health institutions in order to ensure the quality of the data. The internal consistence of the tool was checked and has Cronbach’s α test of 0.89. Supervisors and data collectors received training. Supervisors and data collectors verified the accuracy of the collected data every day. Lastly, Statistical Package for Social Sciences (SPSS) version 26 software was used to analysis the data.

### Data processing and analysis

Data was edited, coded, and entered into Epidata version 3.1 software then, the data was exported to SPSS version 26 software for cleaning and further analysis. Descriptive statistics such as mean, standard deviation, and percentage were determined. The association between the outcome variable and each independent variable was first seen in the binary logistic regression model.

In the second step, independent variables with a *p*-value <0.25 were retained and entered into the binary logistic regression model for multivariable analysis. The degree of association between outcome and independent variables was determined using the odds ratio with a CI of 95% and *p*-value. A *p*-value of <0.05 was considered a cutoff point to declare that there is a statistically significant association between dependent and independent variables. The fitness of the model was tested by Hosmer-Lemeshow goodness-of-fit test, and the value was 0.95.

## Results

### Socio-demographic characteristics of study midwives

In this study, from a sample of 467 midwives, 456 midwives participated, making the response rate 97.64%. Of the study participants, 275 (60.3%) were female. The age of participants ranges from 18 to 54 years, with a mean age of 33.80 (± 3.78 SD) and 250 (54.8%) in union regarding their marital status. Concerning the educational status of study participants, about two-thirds (61.4%) have a degree. Most of the participants, 175 (38.4), were earning 6,163–8,016 in income monthly (Table 1).

**Table 1:** Socio-demographic characteristics of study participants on prevalence of burnout among midwives working public health facilities in West Arsi zone, Ethiopia 2023.

Variables	Categories	Frequency (456)	Percentage (100%)
Sex of study participants	Male	181	39.7
	Female	275	60.3
Age of study participants	18–25	150	32.9
	26–35	127	27.9
	36–44	102	22.4
	≥45	77	16.9
Marital status of study participants	In union	250	54.8
	Not in union	206	45.2
Ethnicity	Oromo	285	62.5
	Ahmara	139	30.5
	Tigre	22	4.8
	Others*	10	2.2
Religion	Orthodox	168	36.8
	Muslim	183	40.1
	Protestant	103	22.6
	Others**	2	0.4
Educational level of study participants	Diploma	117	25.7
	Degree	280	61.4
	Masters and above	59	12.9
Residences	Urban	193	42.3
	Rural	263	57.7
Monthly income (in Ethiopian birr)	≤6,192	112	24.6
	6,163–8,016	175	38.4
	8,017–10,169	127	27.9
	≥10,170	42	9.2

* Gurage, Sidama and Wolayita; ** Adventist.

### Service and organization related characteristics of study participants

About 266 (58.3%) study participants were serving in health centers, and 220 (48.2%) of them gave service for 1–4 years. Regarding work hours, about 217 (47.6%) study participants were in service for more than 40 hours per week. As identified by this finding, about two-thirds (348, or 76.3%) of midwives indicated that their salary was not enough. In addition to this, around 56 percent of study participants were not satisfied with their current job, and 29.8% had symptoms of depression. Furthermore, 45.6% of the study participants had poor social support (Table 2).

**Table 2:** Service and organization related factors on prevalence of burnout among midwives working public health facilities in West Arsi zone, Ethiopia.

Variables	Categories	Frequency (456)	Percentage (100%)
Types of health facilities	Health center	266	58.3
	Primary hospital	147	32.2
	Referral hospital	43	9.4
Year of service	1–4	220	48.2
	5–8	133	29.2
	>8	103	22.6
Work hours per week	≤40Hrs	239	52.4
	>40Hrs	217	47.6
History of chronic medical illness	Yes	18	3.9
	No	438	96.1
Conflict with co-workers	Yes	99	22.1
	No	355	77.9
Availability resource	Yes	297	65.1
	No	159	34.9
Salary is enough to current work	Yes	108	23.7
	No	348	76.3
Conflict with their manager	Yes	235	48.5
	No	221	51.5
Job satisfaction	Satisfied	200	43.9
	Not Satisfied	256	56.1
Depression symptoms	No	320	70.2
	Yes	136	29.8
Social Support	Poor	208	45.6
	Moderate	191	41.9
	Strong	57	12.5

Prevalence of burnout among midwives in working public health facilities in West Arsi zone, Ethiopia 2023.

### Prevalence of burnout among midwives in working public health facilities in West Arsi zone, Ethiopia 2023

**Figure 1:**
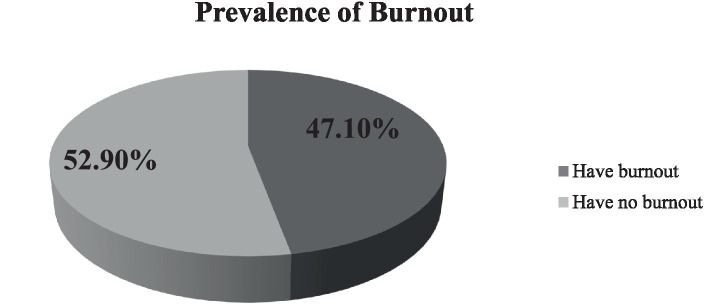
Prevalence of burnout among midwives working in public health facilities in West Arsi zone, Ethiopia 2023.

This study revealed that 47.10% (95% CI: 42.55, 51.75%) of midwives have burnout (Figure 1).

### Factors associated with burnout among midwives working in West Arsi, Ethiopia

Bivariable logistic regression analysis was done with a 95% CI with COR to identify candidate variables, and to control the confounder variables, AOR was calculated in multivariable logistic regression. In binary logistic regression, the variables marital status, educational status, monthly income, working hours per week, conflicts with their metron, social support, job satisfaction, and depression were candidates for multivariable logistic regression. Finally, marital status, working hours per week, conflict with their metron, job satisfaction, and depression fulfilled the criteria of association in the final model.

Marital status is among the factors identified in this study. The study participants who were not in union had a 2.03 times (95% CI: 1.32–3.13) greater chance of developing burnout compared to those in union. The odds of having burnout among midwives working more than 40 h per week were 2.00 times (95% CI: 1.29–3.08) higher compared to their counterparts. In addition, this study identified that midwives who had conflict with their metron were 2.33 times (95% CI: 1.54–3.54) more likely to develop burnout compared to those who had no conflict with their metron. Job satisfaction and depression are also factors identified in this study. The odds of having burnout among midwives who were not satisfied with their current job were 2.39 times (95% CI: 1.56–3.66) greater compared to those satisfied with their current job. Lastly, the odds of developing burnout were 1.71 times (95% CI: 1.06–2.74) higher among midwives who had depression symptoms compared to those who had no symptoms of depression (Table 3).

**Table 3:** Binary and multivariable logistic regression on prevalence of burnout among midwives working public health facilities in West Arsi zone, Ethiopia 2023.

Variables	Categories	Burnout		COR (95% CI)	AOR (95% CI)	*p*-value
		Yes (215)	No (241)			
Marital status of participants	In Union	100 (40%)	150 (60%)	1	1	
	Not in Union	115 (55.8%)	91 (44.2%)	1.90 (1.31–2.75)	2.03 (1.32–3.13)*	**0.01**
Educational status of participants	Diploma	60 (51.3%)	57 (48.7%)	2.05 (1.07–3.93)	1.76 (0.84–3.69)	0.25
	Degree	135 (48.2%)	145 (51.8%)	1.82 (1.01–3.27)	1.85 (0.97–3.51)	0.12
	Masters and above	20 (33.9%)	39 (66.1%)	1	1	
Monthly income	≤6,192	61 (54.5%)	51 (45.5%)	2.15 (1.03–4.48)	1.89 (0.84–4.24)	0.53
	6,193–8,016	84 (48%)	91 (52%)	1.66 (0.83–3.34)	1.64 (0.75–3.61)	0.08
	8,017–10,169	55 (43.3%)	72 (56.7%)	1.74 (0.67–2.83)	1.31 (0.59–2.94)	0.52
	≥10,170	15 (35.7%)	27 (64.3%)	1	1	
Social support	Poor	108 (51.9%)	100 (48.1%)	1.85 (1.01–3.38)	1.80 (0.93–3.49)	0.83
	Moderate	86 (45%)	105 (55%)	1.40 (0.76–2.58)	1.60 (0.81–3.56)	0.35
	Strong	21 (36.8%)	36 (63.2%)	1	1	
Work hours per week	≤40Hrs	91 (41.9%)	126 (58.1%)	1	1	
	>40Hrs	124 (51.9%)	115 (48.1%)	1.49 (1.03–2.16)	2.00 (1.29–3.08)*	**0.03**
Conflict with their metron	No	80 (36.2%)	141 (63.8%)	1	1	
	Yes	135 (57.4%)	100 (42.6%)	2.38 (1.63–3.50)	2.33 (1.54–3.54)*	**0.01**
Job satisfaction	Satisfied	67 (33.5%)	133 (66.5%)	1	1	
	Not satisfied	148 (57.8%)	108 (42.2%)	2.72 (1.85–3.99)	2.39 (1.56–3.66)*	**0.02**
Depression	No	138 (43.1%)	182 (56.9%)	1	1	
	Yes	77 (56.6%)	59 (43.4%)	1.72 (1.15–2.58)	1.71 (1.06–2.74)*	**0.01**

*p-value ≤ 0.05 in multivariable analysis, Hrs. – Hours, 1-Reference group. The bold value is to indicate associated factors.

## Discussion

As identified from this study, the prevalence of burnout among midwives in the West Arsi Zone, Ethiopia, in 2023 was 47.1%, and marital status, working hours per week, conflict with their metron, job satisfaction, and depression were identified as significantly associated factors with burnout. The results of this study suggested that midwives had a high prevalence of burnout, which has a significant impact on the care of the maternity unit. This includes a decline in the quality of care during pregnancy, labor, and the postpartum period, an impact on the maternity continuum of care, a decrease in compassionate maternal care, mistakes with judgment, medical malpractice, and high turnover of staff. Additionally, burnout has an adverse influence on midwives’ personal lives by making them feel disappointed, uninterested in their work, and less engaged with society. The morbidity and mortality rates for mothers and newborns increase as a result of those circumstances.

The finding of this study is in line with the study conducted in Eastern Amhara, Ethiopia (20), which was 51.9%. Comparably, the results of this survey align with a study that was done among healthcare providers in Mekelle City, Ethiopia (37), which was 47.5%. This could be because those investigations were carried out in comparable settings within the same nation. The result of this study is also consistent with the findings of the systemic review and meta-analysis (12) and the study conducted in Germany (38), which were 48.3 and 50%, respectively. This may be because midwives are currently underpaid and have a heavy workload at their positions across the globe. The National Health Service (NHS) data on midwife burnout further supports this, showing that almost three-quarters (64%) of midwives feel fatigued or burned out at the end of all or most of their working shifts (39).

The result of this study is higher than the studies conducted in Dutch (15), Western Canadian midwives (40), and Norwegian midwives (7), which were 7, 34.7 and 20%, respectively. One explanation for this might be because, as is the situation in developing countries like Ethiopia, midwives operate in environments without the basic clinical supplies required to provide care both during and after the birth process. This caused the midwives to worry that they would get infectious diseases. They were unable to complete their work to a high enough standard when they lacked the necessary resources, which increased their risk of burnout. Another explanation could be that midwives in developing nations have higher workloads and less access to training, compensation, and educational opportunities than in wealthy nations.

On the other hand, the finding of this study was higher than those of the studies conducted among health-care professionals working at Gondar University Hospital, Ethiopia (41), nurses working in Ethiopia (42), Debre Berhan University medical students (43), and Dessie, Ethiopia (44) which were 13.7, 39, 34.0%, and 15.1%, respectively. This might be due to the fact that the former studies were conducted among all health care providers in a single institution. Additionally, the possible discrepancies might be due to variations in study duration.

However, the finding of this study is lower than the study conducted on burnout among maternity providers in Northern Ghana (14) which was 65.8%. Similarly, the findings of this study were lower than those of the study conducted among health professionals in Dire Dawa City, Eastern Ethiopia (45), and a tertiary teaching hospital in Tanzania (46) which were 54.1 and 62%, respectively. This may be because our study was conducted among midwives, whereas the previous one was conducted among all healthcare professionals involved in maternity services. Furthermore, this might be due to the studies conducted in different settings. Another possible reason is that the former study conducted in Dire Dawa, Ethiopia, was conducted during COVID-19, which might increase the case.

One of the factors found in this study is marital status. Compared to midwives in unions, those who were not in one have a higher risk of experiencing burnout. The researches done in Gonder, Ethiopia (41), and Ghana (47) lends credence to this. One possible reason for this outcome is that sharing responsible, fruitful, and fulfilling lives is facilitated by a strong family and relationship.

The results of this study showed that midwives who worked more than 40 hours a week had a higher risk of experiencing burnout. This is supported by the studies conducted in Taiwan (48) and Germany (49). This might be due to the fact that extended work hours would reduce employees’ free time and leave them with little opportunity for rest and relaxation, which would be detrimental to their wellbeing. Long work hours may also be harmful to wellbeing since they can cause sleep deprivation and stimulation of the hypothalamic–pituitary–adrenal axis (HPAA). This results in stress, anxiety, and depression, all of which have detrimental effects on a person’s behavior and physiology.

Another aspect this study uncovered is a conflict with a metron. Compared to participants who did not have disagreements with their metron, those who did had a higher likelihood of experiencing burnout symptoms. The studies carried out in Ethiopia’s Eastern Amhara provide (20) a systematic review of burnout in sub-Saharan Africa (50), in West Canada (40) and Swedish midwives (51) evidence in favor of this. This might be the result of a confrontation between the team leader and employees, which would raise tension and lower motivation to work, causing interruptions at work, lower productivity, absenteeism, turnover, stress, and termination.

Additionally, this study showed a strong correlation between burnout and job satisfaction. Compared to midwives who were satisfied with their jobs, those who were not were more likely to suffer from burnout. This is supported by the studies conducted in Croatia (52), Brazil (53), and Iran (54). Potentially due to the fact that happy workers need fulfilling jobs. It encompasses a variety of aspects of an individual’s experience, including their perception of their place of employment, the tasks they have to perform every day, the part management plays, and their interactions with other employees.

Lastly, burnout symptoms were more common among midwives with depression symptoms than they were among those without. Studies carried out in Saudi Arabia (55) and China (56) support of this. This could be because depression and burnout can have physical symptoms that are similar.

## Limitations

One of the study’s limitations was that it lacked questions for participants to provide information in order to tackle burnout. Next to this, due to the nature of a cross-sectional study, it is difficult to establish a causal relationship between risk factors and burnout. In addition to this, it has social desirability bias because the study participants may exaggerate the report by thinking as they gain some comprehension. However, to decrease this bias, data collectors clearly described the purpose of the study during data collection. Furthermore, there are a few studies found in Ethiopia, specifically among midwives, so it challenges us to get adequate literature.

## Conclusion

This study found that in the study area, almost half of the midwives experienced burnout. Burnout was significantly correlated with a number of factors, including not being married, working over 40 hours a week, having conflict with their mentor, not being satisfied with their job, and experiencing symptoms of depression.

## Recommendations

It is recommended that midwives develop respectful interactions with both their mentors and colleagues. Additionally, midwives should be happy in their work, not because of incentives or pride, but because of the care they provide, saving mothers and newborns one at a time. Secondly, we suggest that zonal health offices set up systems that improve working hours and boost job satisfaction by developing various incentive programs that raise wages for health personnel, offer a variety of rewards, and regularly monitor employees’ issues.

Thirdly, governments and policymakers should recognize that midwives, who experience burnout may find it difficult to make clinical decisions, communicate effectively with patients and colleagues, handle pressure from the workplace, and ultimately provide lower-quality care and worse patient outcomes. Thus, they should conduct regular evidence-based assessments regarding the needs of midwives in terms of working hours, financial problems, health problems, interaction with their colleagues, and appropriate communication on their payment. In addition to this, governments and policymakers should work to construct attractive recreational environments near their work. Further, providing regular psychotherapy for midwives might be a better way to tackle mental health problems, including burnout. Finally, we recommend to upcoming researchers that it is better if the topic is supported by a qualitative method as well as if it assesses strategies to tackle burnout.

## Data availability statement

The original contributions presented in the study are included in the article/supplementary material, further inquiries can be directed to the corresponding author.

## Ethics statement

The studies involving humans were approved by the Madda Walabu University, Sheshemene Campus School of Health Science Ethical Review Committee. The studies were conducted in accordance with the local legislation and institutional requirements. The participants provided their written informed consent to participate in this study.

## Author contributions

SA: Conceptualization, Data curation, Formal analysis, Funding acquisition, Investigation, Methodology, Project administration, Resources, Software, Supervision, Validation, Visualization, Writing – original draft, Writing – review & editing. MH: Conceptualization, Data curation, Formal analysis, Funding acquisition, Investigation, Methodology, Project administration, Resources, Software, Supervision, Validation, Visualization, Writing – original draft, Writing – review & editing. MA: Conceptualization, Data curation, Formal analysis, Funding acquisition, Investigation, Methodology, Project administration, Resources, Software, Supervision, Validation, Visualization, Writing – original draft, Writing – review & editing. FH: Conceptualization, Data curation, Formal analysis, Funding acquisition, Investigation, Methodology, Project administration, Resources, Software, Supervision, Validation, Visualization, Writing – original draft, Writing – review & editing. HG: Conceptualization, Data curation, Formal analysis, Funding acquisition, Investigation, Methodology, Project administration, Resources, Software, Supervision, Validation, Visualization, Writing – original draft, Writing – review & editing. ST: Conceptualization, Data curation, Formal analysis, Funding acquisition, Investigation, Methodology, Project administration, Resources, Software, Supervision, Validation, Visualization, Writing – original draft, Writing – review & editing. DY: Conceptualization, Data curation, Formal analysis, Funding acquisition, Investigation, Methodology, Project administration, Resources, Software, Supervision, Validation, Visualization, Writing – original draft, Writing – review & editing. LW: Conceptualization, Data curation, Formal analysis, Funding acquisition, Investigation, Methodology, Project administration, Resources, Software, Supervision, Validation, Visualization, Writing – original draft, Writing – review & editing.
